# Microbial enzymatic indices for predicting composting quality of recalcitrant lignocellulosic substrates

**DOI:** 10.3389/fmicb.2024.1423728

**Published:** 2024-11-11

**Authors:** Loubna El Fels, Ahmed Naylo, Martin Jemo, Nidal Zrikam, Ali Boularbah, Yedir Ouhdouch, Mohamed Hafidi

**Affiliations:** ^1^Laboratory of Microbial Biotechnologies, Agrosciences and Environment (BioMAgE), Labelled Research Unit-CNRST N°4, Faculty of Sciences Semlalia, University Cadi Ayyad (UCA), Marrakech, Morocco; ^2^Laboratoire Bioressources et Sécurité Sanitaire des Aliments, Faculté des Sciences et Techniques, Université Cadi Ayyad, Marrakech, Morocco; ^3^AgroBiosciences Program, College for Sustainable Agriculture and Environmental Sciences, University Mohammed VI Polytechnic (UM6P), Ben Guerir, Morocco; ^4^Center of Excellence for Soil and Fertilizer Research in Africa, College of Agriculture and Environmental Sciences, Mohammed VI Polytechnic University, Ben Guerir, Morocco; ^5^African Sustainable Agriculture Research Institute (ASARI), College for Sustainable Agriculture and Environmental Sciences, University Mohammed VI Polytechnic (UM6P), Laayoune, Morocco

**Keywords:** lignocellulosic-sludge composting, microbial enzymes index, microbial dynamic, maturity index, alkaline pohosphatase

## Abstract

Three different enzymes alkaline phosphatase, Urease and Dehydrogenase were measured during this study to monitor the organic matter dynamics during semi-industrial composting of mixture A with 1/3 sludge+2/3 palm waste and mixture B with ½ sludge+1/2 palm waste. The phosphatase activity was higher for Mix-A (398.7 µg PNP g^−1^ h^−1^) than Mix-B (265.3 µg PNP g^−1^ h^−1^), while Mix-B (103.3 µg TPF g^−1^d^−1^) exhibited greater dehydrogenase content than Mix-A (72.3 µg TPF g^−1^ d^−1^). That could contribute to the dynamic change of microbial activity together with high amounts of carbonaceous substrates incorporated with the lignocellulosic. The gradual increase in the dehydrogenase from the compost Mix-A implies that high lignocellulosic substrate requires gradual buildup of dehydrogenase activity to turn the waste into mature compost. A higher pick of urease with a maximum activity of 151.5 and 122.4 µg NH_4_-N g^−1^ h^−1^ were reported, respectively for Mix-A and B. Temperature and pH could also influence the expression of enzyme activity during composting. The machine learning well predicted the compost quality based on NH_3_/NO_3_, C/N ratio, decomposition rate and, humification index (HI). The root mean square error (RMSE) values were 1.98, 1.95, 4.61%, and 4.1 for NH^+^_3_/NO^−^_3_, C/N ratio, decomposition rate, and HI, respectively. The coefficient of determination between observed and predicted values were 0.87, 0.93, 0.89, and 0.94, for the r NH_3_/NO_3_, C/N ratio, decomposition rate, and HI. Urease activity significantly predicted the C/N ratio and HI only. The profile of enzymatic activity is tightly linked to the physico-chemical properties, proportion of lignocellulosic-composted substrates. Enzymatic activity assessment provides a simple and rapid measurement of the biological activity adding understunding of organic matter transformation during sludge-lignocellulosic composting.

## 1 Introduction

Composting offers a promising avenue for valorizing organic waste, which is a rich source of nutrients for plant fertilization and contributes to carbon sequestration, accounting for environmental sustainability (Fernández-Delgado et al., 2020). Composting represents an efficient way to enrich soil in which organic wastes can be recycled to obtain organic fertilizers utilized in agricultural fields (Finore et al., 2023). Their incorporation into the soil as organic matter or solid waste compost promotes microbiological activity and diversity that are prime constituents of the soil environment, which is accountable for driving the nutrient cycle and energy transfer (Nannipieri et al., 2018; Megha and Simpy, 2014). This is accomplished through the activity of enzymes (Kong et al., 2009; Chen et al., 2020). The soil microbial biomass is related to the soil organic matter content as well as its origin. Bastida et al. (2008) showed that enzymatic activities are very sensitive indicators of changes related to soil management practices, climatic variations, and contaminations, which is why they have been considered in the establishment of agronomic and non-agronomic indices of soil quality. The most important general indicators of soil microbial activity are microbial biomass C. Microbial communities in the soil are enhanced and stimulated by the addition of organic waste and readily available nutrients and C compounds (Vinhal-Freitas et al., 2010).

Although several studies have shown that compost can improve soil by promoting appropriate biological activity. Thus, microbial biomass, being the living part of compost organic matter, can be a good index for comparing compost maturity (El Fels et al., 2014a, 2015). During composting, organic matter is transformed into a rich humic product by the action of microorganisms and their enzymes (Vargas-García et al., 2010).

However, achieving a high standard of compost is hindered by several factors such as the carbon-to-nitrogen ratio (C: N) (Zhang et al., 2020), improper temperature fluctuations can impact compost quality (Zhao et al., 2021), as well as the essential interactions between microbial communities, organic material, and the enzymatic processes governing their degradation during the thermophilic and maturation phases (Mandpe et al., 2019).

It has been shown that the moisture level of the material approximately 60% contributes to increased microbial activity. The bulking agent improves oxygenation inside the composting pile (Moreno et al., 2007). As a consequence, the influence of enzymatic activities on the composting progress has long been the subject of several studies (Bremner and Mulvaney, 1978; Hemati et al., 2021; Ulmer et al., 1984).

It is also well known that several biochemical reactions are catalyzed by enzymes during composting. For instance, the mineralization of organic N during composting, which involves the release of N from non-peptide C-N bonds in amino acids and urea, is mediated by amidohydrolases and dehydrogenases (Tabatabai, 1994).

Various categories of enzymes including *ß*-glucosidase, phosphatase, amylase, xylanase, urease, and protease are produced also by the different groups of microbes to degrade the organic molecules (Hemati et al., 2021). During the composting thermophilic phase, increasing enzymatic activities from the resident microbes accelerate to breaking down of organic compounds, ameliorating qualitatively and quantitatively the properties of compost (Xu et al., 2019). Phosphatase and urease are well-known enzymes for transforming organics and improving nutrient cycling in soil nutrients (Kumar et al., 2020). Urease enzyme activity is vital to understand the process of nitrogen mineralization (Sudkolai and Nourbakhsh, 2017; Kumar et al., 2020). Enzyme activity is essential in both mineralization and transformation of organic carbon and plant nutrients. These above-mentioned enzymes are produced by the resident microorganisms during the composting process; thus, their activity is important to determine the degradation process and quality of the composting.

Green residues and wood are important proportions of wastes valorized for composting, and it is highly rich in lignocellulose (cellulose, hemicellulose, and lignin) compounds. Tiquia et al. (2002) showed a significant positive correlation with microbial population and 19 examined enzymes. The population of fungi and actinomycetes (microorganisms active in the degradation of cellulose, hemicellulose, and lignin) were positively correlated with esterase, valine amino-peptidase, *α*-galactosidase, *β*-glucosidase, and lipase. Lignin can be mineralized by fungi strains (Hemati et al., 2021). However, during the thermophilic phase, most fungi are eliminated in profit of actinomycetes, which are only slow-decomposer of organic compounds and make the decomposition process longer (Hemati et al., 2021). Therefore, the identification of microbes resistant to thermophilic conditions and capable of mineralizing lignin compounds or maintaining greater enzymatic activities during the maturation stage will improve the compost quantitative and qualitatively. Given the importance of enzymatic activity, the activity of some major classes of catalytic enzymes (extracellular or intracellular) is sometimes proposed as a compost quality sensor. The effect of physicochemical parameters on enzymatic activity has been developed (Wu et al., 2017). However, the direct effect of lignocellulosic material is still not yet addressed. Enzymes, free or adsorbed, have a lifetime and activity that vary widely depending on the several physicochemical and biological properties occurring during composting. To understand the interaction of enzymes and their behavior taking place over the sludge lignocellulosic waste during semi-industrial composting, this study aimed to assess the variation trends and the activity of the main enzymes alkaline phosphatase, urease, and dehydrogenase with different proportions of lignocellulosic substrate for the best prediction of the compost quality.

## 2 Materials and methods

### 2.1 Materials

Sewage sludge and palm organic wastes are the two substrates used during composting. The sewage sludge comes from an activated sludge wastewater treatment plant in Marrakech urban council. The date palm, leaves, and stems were chopped in 5 cm. The tree material was collected from the urban waste of the palm forest of Marrakech city.

### 2.2 Composting preparation, sampling, and physicochemical analysis

The composting process was conducted on a composting platform. Two mixture (Mix) treatments were as follows: Mix-A was prepared with ^1^/^2^ sludge and ^2^/^3^ palm tree waste with a total volume of 4 m^3^. Mix-B was prepared at the sludge and tree palm waste ratio of half for each with a total volume of 4 m^3^. The Mix-A and Mix-B have the NH_4_^+^/NO_3_^−^ composition of 13.75 ± 0.27 and 15. 6 ± 0.11, respectively. The starting C/N ratio was 26.2 ± 0.42 for Mix-A and 27.4 ± 0.18 for Mix-B. The decomposition rate was almost similar for Mix-A and Mix-B, while the HI was higher (24.53 ± 0.19) than for Mix-B (15.38 ± 0.18). The moisture content during composting was adjusted to 60%. To enable mixture ventilation, composting windrows were turned over manually with weekly frequency. The temperature of the windrow was daily recorded using sensors with data memory (PH0700115 model 1.20, Ector-Trac, ability software, ECTOR France). The two treatments were composted for 13 weeks. The final moisture content was determined by drying the compost sample at 105°C for 48 h. The total organic carbon and ash content of dried samples were calculated after calcination at 600°C for 6 h. TKN was assayed by using the classical Kjeldahl procedure according to AFNOR T90-1110 standard, and Phosphorus by Olsen method. The high-temperature level reached during composting was approximately 65°C (thermophilic phase). This is the result of microbial activity resulting from the degradation of the simple molecules.

### 2.3 Microbial dynamic assessment

Microorganism enumeration was carried out on composting time extracts agar (CTEA) prepared as follows: One liter of distilled water and 35 g of composting time sample were mixed overnight to prepare composting time extract agar (CTEA). Agar (15 g) was added to the filtrate collected after it had been filtered and sterilized at 120°C for 15 min. 0.1 mL of composting diluted samples from 10^−2^ to 10^−6^ were spread over the surface of the prepared CTEA. The plates were incubated at 28°C for mesophilic and 45°C for thermophilic microflora enumeration according to El Fels et al. (2014b, 2015).

### 2.4 Enzymatic activities

#### 2.4.1 Dehydrogenase activity

The dehydrogenase activity was measured by the quantification of the triphenylformazan (TPF) obtained after the incubation of each composting stage with triphenyltetrazolium chloride (TTC) as substrate (Thalmann, 1968); 5 g of fresh composting substrate was well mixed with 5 mL of TTC solution (0.8% in (0.1 M) Tris–HCl buffer (pH 7.6)) and incubated at 30°C for 24 h. The TPF produced was extracted with acetone (90%) and the optical density was measured against the blank at 546 nm. The blank was obtained by mixing 5 g of fresh composting substrate with 5 mL of (0.1 M) Tris–HCl buffer (pH 7.6) and incubated in the same condition as in the assay. The amount of TPF obtained was determined by using a standard curve with concentrations varying between 0 and 40 μg TPF mL^1^.

#### 2.4.2 Alkaline phosphatase activity

The alkaline phosphatase activity was measured by the determination of *p*-nitrophenol (PNP) released after incubation of composting substrate with *p*-nitrophenyl phosphate (Eivazi and Tabatabai, 1977). A quantity of 1 g of composting substrate was mixed with 4 mL of modified universal buffer solution and 1 mL of 5.85% of *p*-nitrophenyl phosphate and incubated for 1 h at 37°C with shaking. The reaction was stopped by adding 1 mL of CaCl_2_ (0.5 M) and 4 mL of NaOH (0.5 M). The optical density of the supernatant was measured at 400 nm. The blank was performed by adding 1 mL of p-nitrophenyl after the addition of 1 mL of CaCl_2_ and 4 mL of NaOH. The amount of PNP was determined by using a standard curve with concentrations ranging between 0 and 20 μg PNP mL^−1^.

#### 2.4.3 Urease activity

The urease activity was measured according to Tabatabai and Bremner’s (1972) methodology by the determination of the amount of ammonium produced after the incubation of composting substrate with urea; 5 g of fresh composting substrate was mixed with 2.5 mL of urea solution (0.08 M) and incubated for 2 h at 37°C with shaking. The reaction was stopped by adding 50 mL of KCL (1 M). After 30 min of incubation with shacking, the ammonium content was determined spectrophotometrically at 690 nm. A blank was made by adding 2.5 mL of distilled water and adding urea solution at the end of the reaction and immediately before KCL addition. The amount of NH_4_-N was determined by using a standard curve with concentrations varying between 0 and 2.5 μg NH_4_-N mL^−1^.

### 2.5 Data modeling, the linear mixed regression model

The linear mixed-effect model accounts for sample size imbalances and the confounding effects of uncontrolled studies. The NH_4_^+^/NO_3_^−^ and C/N ratios, decomposition rate, and humic index (HI) variables were modeled in two major components using the “*lm*” function in the R package (RStudio Team, 2024). The linear function with a fixed-effect trend “f (i)” with the *^i^* th level of the explanatory variables (composting stage, compost mixtures, phosphatase, dehydrogenases, urease activity, bacteria, fungi, and actinobacteria compositions) were constructed to predict NH_4_^+^/NO_3_^−^ and C/N ratios, decomposition rate, and HI as response variables from linear mixed models in R (RStudio Team, 2024). The “*lm*” was fitted to the trained datasets using a train control to predict the datasets. The predicted against the observed values were visualized in a Scatterplot graph.

### 2.6 Statistical analysis

The descriptive and general statistical analyses were conducted for the aggregated datasets using the JMP software (JMP, 2022). The effect sizes were examined for suitability for analysis using the Shapiro–Wilk and Levene’s tests. Multiway analysis of variance (MANOVA) was conducted to assess the effect of the imposed treatment on compost quality change using the JMP software (JMP, 2022). Fisher’s test was used to separate means that were different at *p* < 0.05 when the Fischer (*F*) value was significant from the ANOVA (*p* < 0.05). Significance levels are given by >0.05 (ns), * at *p* < 0.05, ** at *p* < 0.01, and *** at *p* < 0.001.

## 3 Results

### 3.1 Percentage of microbes decomposing recalcitrant lignocellulosic substrate

**Figure 1:**
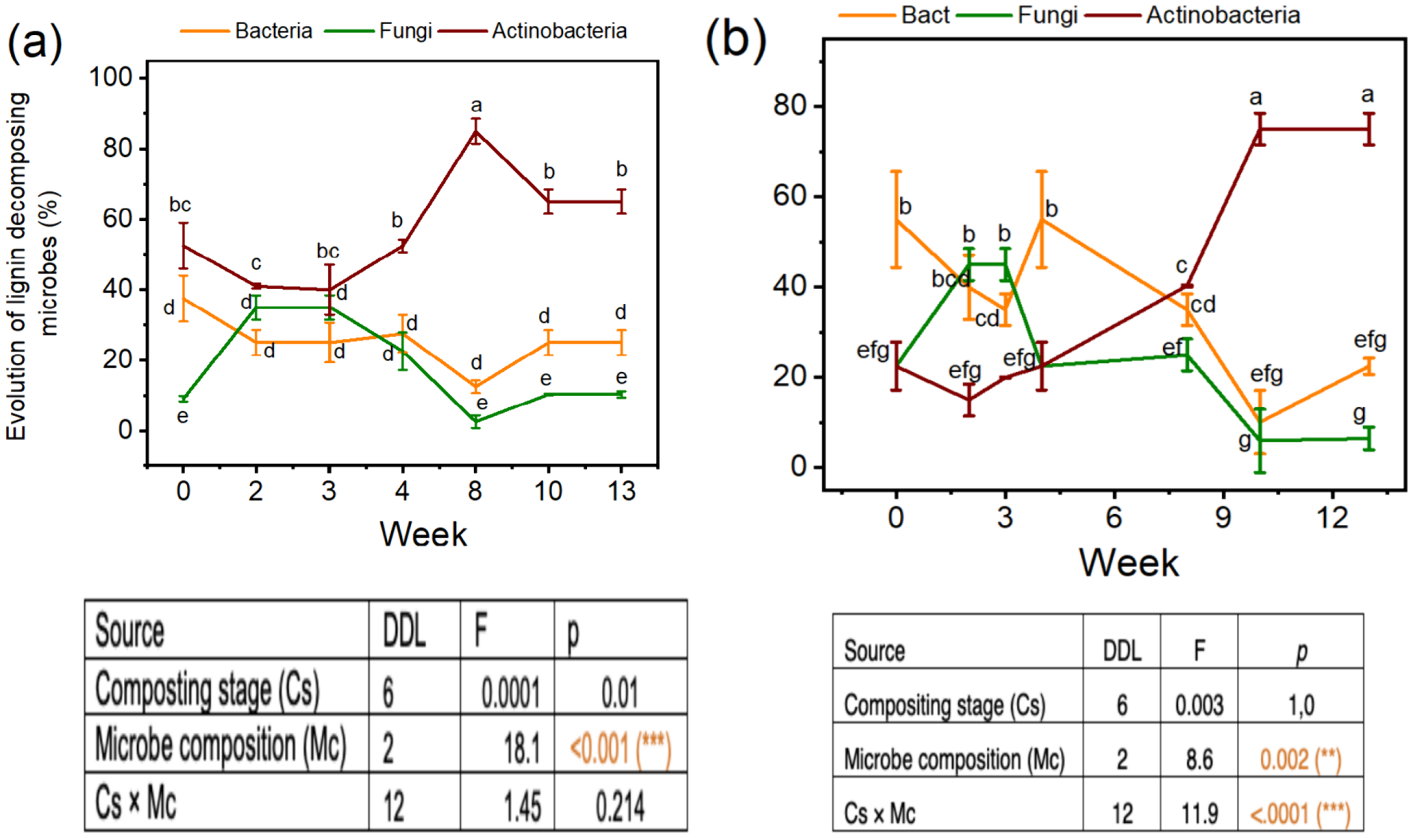
Microbial dynamic during composting of Mix-A (a) and Mix-B (b).

Analysis of variance probabilities effects for the evolution in microbial compositions, especially the bacteria, fungi, and actinobacteria in the compost mixture and composting times are reported in Table 1. The compost mixture had a significant effect at *p* < 0.05 on the bacteria and actinobacteria, respectively (Table 1). Similarly, the effects of composting times on the evolution of bacteria, fungi, and actinobacteria percentages were significant at *p* < 0.05 (Table 1). Thermophilic fungal microflora increased by 40% during the thermophilic stage. However, the functional actinobacteria group presented a peak during the maturation stage of 80–90%, especially for mixture A. Actinobacteria composition was always higher than bacteria and fungi composition for Mix-A during the different composting times that increased during the maturation than thermophilic stages, and the microbe composition effect was highly significant (Figure 1a).

**Table 1:** ANOVA analysis of the effects of composting stage, mixture, and time—and their interactions—on phosphatase, dehydrogenase, urease activities, and the composition of bacteria, fungi, and actinobacteria during composting.

	Phosphatase activity (μg PNP g^−1^ h^−1^)		Dehydrogenase activity (μg TPF g^−1^d^−1^)		Urease activity (μg NH_4_-N g^−1^ h^−1^)		Bacteria compositio*n* (%)		Fungi composition (%)		Actinobacteria composition (%)	
Source	*F*	*p*-value	*F*	*P*-value	*F*	*p*-value	*F*	*p*-value	*F*	*p*-value	*F*	*p*-value
Composting stage (Cs)	0.03	0.86	0.02	0.90	0.20	0.66	0.09	0.76	0.2	0.7	0.0	1.0
Compost mixture (Cm)	**8.38**	0.009 (**)	**11.88**	0.003 (**)	0.31	0.58	**4.04**	**0.06**	1.7	0.2	**8.3**	0.009 (**)
Composting time (Ct)	2.33	0.14	2.58	0.12	0.05	0.83	**6.17**	0.02 (*)	**12.8**	0.002 (*)	**26.1**	<0.001 (***)
Cs × Cm	0.06	0.80	0.04	0.85	0.08	0.78	2.71	0.12	0.0	0.9	2.0	0.2
Cs × Ct	0.57	0.46	0.31	0.58	0.29	0.60	0.01	0.98	0.6	0.4	0.4	0.5
Cm × Ct	0.58	0.46	**26.10**	<0.001 (***)	0.07	0.80	1.74	0.20	0.9	0.4	3.8	0.1
Cs × Cm × Ct	0.18	0.68	0.66	0.43	0.02	0.90	1.96	0.18	0.4	0.5	0.7	0.4

Bold numbers are significant at the *t*-test: *p* < 0.05.

We noticed a decrease in the bacteria and fungi percentages during the maturation stages in the Mix-A (Figure 1a) to reach 20 and 10% for mesophilic and 30and 10% for thermophilic microflora, respectively. About Mix-B, there was a higher number of bacteria (70%) than actinobacteria (15%) and fungi (15%) at the thermophilic stage of composting, but the amount decreased in the maturation stage (Figure 1b). Contrary to thermophilic bacteria, there was a gradual build-up in the thermophilic actinobacteria population that reached higher concentrations during the maturation stages approximately 90 and 80%, corresponding to the 10 and 13 weeks of compositing for mix A and B, respectively (Figure 1b). Composition and the interaction between microbe composition and composting stage were significant at *p* < 0.05 (Figure 1b).

### 3.2 Enzyme concentrations and microbial activities in the compost mixtures

**Figure 2:**
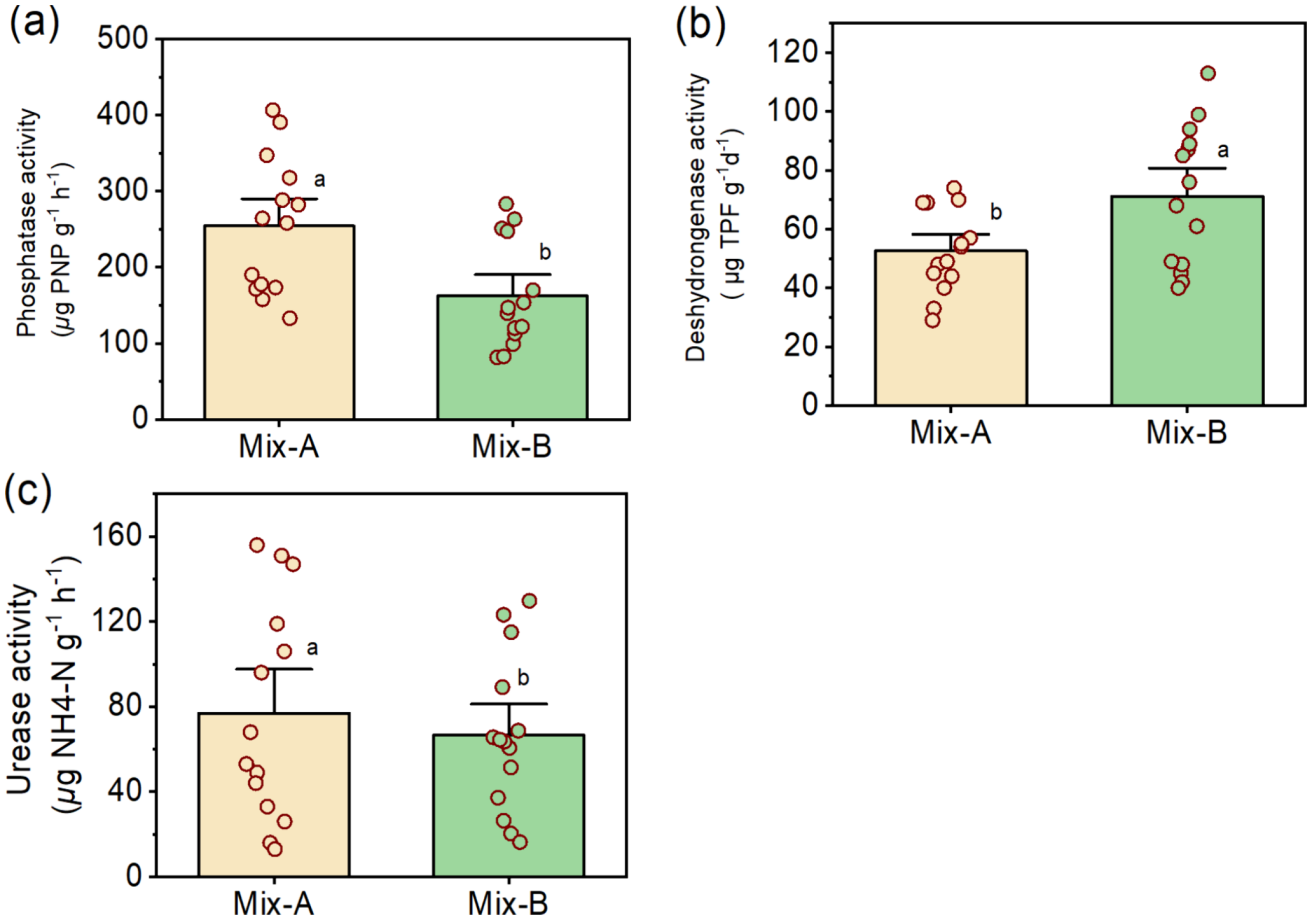
Enzymes amount phosphatase activity (a), urease activity (b), and dehydrogenase activity (c) for Mix-A and Mix-B.

Table 1 presents the analysis of the variance of probabilities of phosphatase, dehydrogenase, and urease concentrations measured in the compost Mix-A and Mix-B. Phosphatase content was higher in Mix-A than in Mix-B, while the dehydrogenase concentration was greater in Mix-B than in Mix-A (Figures 2a,b). In contrast with phosphatase and dehydrogenase concentrations, urease concentrations were not significantly different from each other between the compost mixtures (Figure 2c).

**Figure 3:**
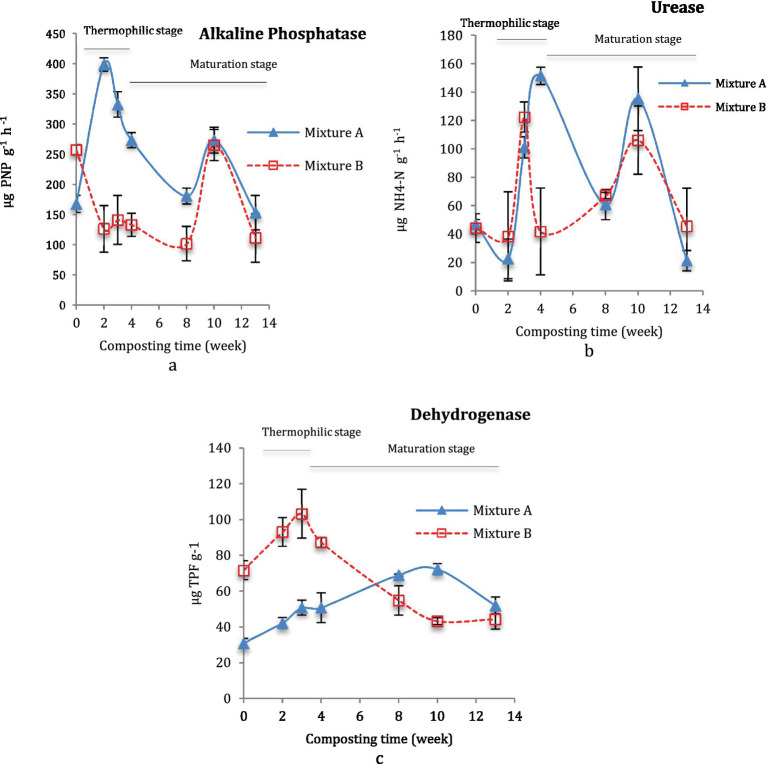
Enzymatic activity during composting of mixture A and B: Phosphatase (a), urease (b), and dehydrogenase (c) during composting (b).

Figure 3 displays the activities of phosphatase, dehydrogenase, and urease in the decomposition of Mix-A and Mix-B during the thermophilic and maturation stages of composting. A higher pick of phosphatase activity was noted for the Mix-A (398.7 μg PNP g^−1^ h^−1^) at 2 weeks of incubation during the thermophilic phase, and another high pick in the maturation stage at 10 weeks with 273.43 μg PNP g^−1^ h^−1^. The phosphatase activity was higher in Mix-A than the Mix-B, which showed only one pick with 265.35 μg PNP g^−1^ h^−1^ at 10 weeks (Figure 3a). Similarly, Mix-A displayed higher picks of urease enzymes at 3 and 10 weeks than Mix-B (Figure 3b). Two important picks, one at the thermophilic stage from at 3 weeks, with a maximum activity of 151.59 and 122.42 μg NH_4_-N g^−1^ h^−1^, and the second pick at 10 weeks of composting with a maximum of approximately 135.26 and 106.19 μg NH_4_-N g^−1^, were reported, respectively, for Mix-A and B (Figure 3b).

The dehydrogenase activity was significantly higher during the thermophilic stage (103.28 μg TPF g^−1^ d^−1^) and significantly decreased during the maturation stage for the Mix-B (Figure 3c). On the contrary, a lower dehydrogenase activity was observed at an early stage of composting the thermophilic stage for Mix-A then showed a continuous upward tendency until the 10^th^ week (maturation stage) to reach a peak of approximately 72.29 (Figure 3c).

### 3.3 Physicochemical index to predict compost quality

Table 2 presents the results of the analysis of variance probabilities, or the effects of composting, stage, moistures, and timing of composting on the NH_4_^+^/NO_3_^−^, C/N ratios, the decomposition rate, and HI, which indicator of compost maturation and quality.

**Table 2:** Analysis of variance (ANOVA) probabilities for the effects of the composting stage, mixtures times, and their interactions on NH_4_^+^/NO_3_^−^ and C/N ratios, decomposition rate, and HI in the composting process.

Source	NH4+/NO3-ratio		C/N ratio		Decomposition rate (%)		HI (%)	
	*F*	*p*-value	*F*	*p*-value	*F*	*p*-value	*F*	*p*-value
Composting stage (Cs)	0.000	1.00	0.00	1.00	0.00	1.00	0.00	1.00
Compost mixture (Cm)	0.001	0.97	1.19	0.29	0.38	0.55	**17.74**	<0.001 (***)
Composting time (Ct)	**45.8**	<0.001 (***)	**203.9**	<0.001 (***)	**62.61**	<0.001 (***)	**246.9**	<0.001 (***)
Cs × Cm	0.00	1.00	0.00	1.00	0.00	1.00	0.00	1.00
Cs × Ct	0.00	1.00	0.00	1.00	0.00	1.00	0.00	1.00
Cm × Ct	0.02	0.90	0.24	0.63	0.01	0.94	0.26	0.62
Cs × Cm × Ct	0.00	1.00	0.00	1.00	0.00	1.00	0.00	1.00

Bold numbers are significant at the *t*-test: *P* < 0.05.

The composting time effects were highly significant on the NH_4_^+^/NO_3_^−^, C/N ratios, the decomposition rate, and HI, disclosing lower values of NH_4_^+^/NO_3_^−^, and C/N in the 10 and 13 weeks of composting stages compared to 0, 2, and 4 weeks for the Mix-A and Mix-B (Table 3).

**Table 3:** NH_4_^+^/NO_3_^−^, and C/N ratios, decomposition rate, and HI of the compost mixtures in the different composting stages.

Composting stage	NH4+/NO3-ratio		CN ratio		Decomposition rate		HI ratio	
	Mix-A	Mix-B	Mix-A	Mix-B	Mix-A	Mix-B	Mix-A	Mix-B
Week 0	13.4 ± 0.27	15.8 ± 0.11	25.6 ± 0.42	27.7 ± 0.18	1.1 ± 0.07	1.3 ± 0.18	24.3 ± 019	15.6 ± 0.18
Week 2	7.3 ± 0.21	6.6 ± 0.16	27.2 ± 0.03	27.7 ± 0.13	21.4 ± 0.07	20.6 ± 0.23	30.2 ± 0.15	23.2 ± 0.03
Week 3	4.7 ± 0.07	4.6 ± 0.19	20.8 ± 0.11	23.7 ± 0.11	27.6 ± 0.25	24.5 ± 0.06	36.2 ± 0.14	32.1 ± 0.03
Week 4	3.7 ± 0.18	2.7 ± 0.15	19.2 ± 0.14	20.5 ± 0.23	29.5 ± 0.21	27.8 ± 0.07	40.3 ± 0.13	35.4 ± 0.14
Week 8	1.2 ± 0.001	1.4 ± 0.10	12.4 ± 0.28	12.9 ± 0.07	33.3 ± 0.21	29.4 ± 0.29	47.4 ± 0.14	45.4 ± 0.14
Week 10	0.3 ± 0.002	0.2 ± 0.17	10.6 ± 0.07	11.5 ± 0.11	36.2 ± 0.03	34.6 ± 0.55	52.3 ± 0.21	50.8 ± 0.03
Week 13	0.1 ± 0.001	0.2 ± 0.02	10.3 ± 0.21	10.1 ± 0.003	41.1 ± 0.05	41.4 ± 0.31	63.7 ± 0.07	48.5 ± 0.27

In contrast to NH_4_^+^/NO_3_^−^, and C/N ratios, the decomposition rate and HI, values were greater in the latter stages (weeks 10 and 13) of composting than in weeks 0, 2, and 4 for the Mix-A and Mix-B (Table 3). The ANOVA effect testing the composition mixture on the HI was significant, and the average HI for Mix-A was higher than for Mix-B.

**Figure 4:**
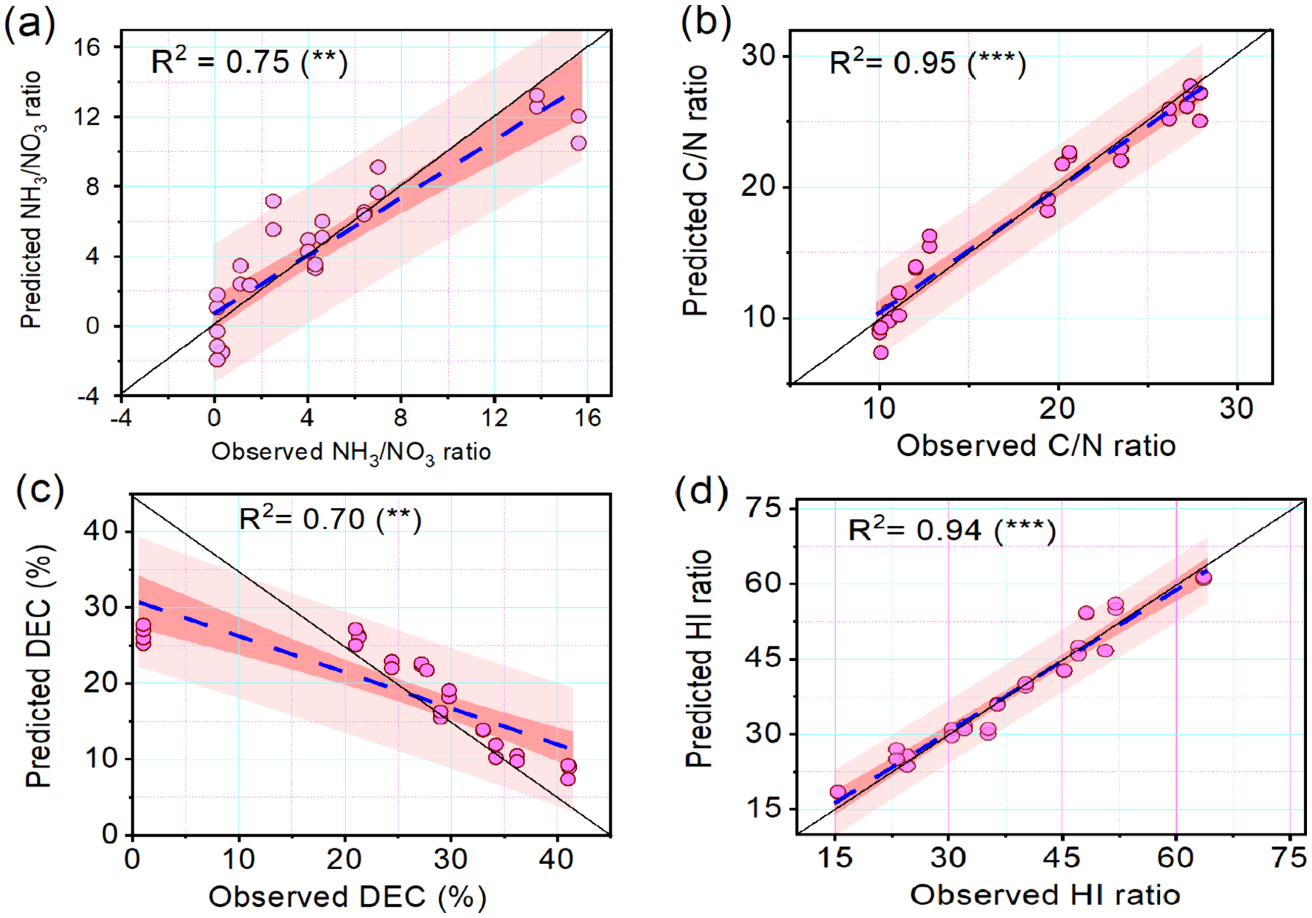
Scatterplots showing the relationship between observed and predicted **(A)** NH 3 /NO 3, **(B)** C/N ratio, **(C)** decomposition rate (DEC), and **(D)** humification index (HI) of the compost mixtures. The trained coefficient of determination (R 2) is indicated. Levels of significance are indicated by **for significance at *p* < 0.01 and highly significant at *p* < 0.001, respectively.

**Figure 5:**
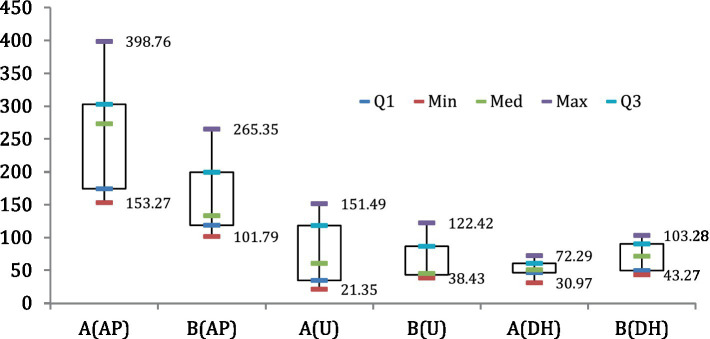
Box and whiskers plot of enzymes profile during composting of mixture A and B.

The relationship between the predicted and observed variables was visualized in the scatter plots (Figure 4). The coefficient of determination (*R*^2^) between the observed and predicted NH_4_^+^/NO_3_^−^, was 0.75 (Figure 4a), while the *R*^2^ between observed and predicted C/N ratio was 0.95 (Figure 4b). Regarding the decomposition rate and HI, the correlation coefficient between the observed and predicted decomposition rates was 0.75 (Figure 4c), while the relationship between the predicted and observed HI was very strong (0.90) (Figure 4d). The whisker plot (Figure 5) showed the different trends in microbial dynamics based on enzymatic activities during the composting of Mix-A and Mix-B. The results showed that the most important enzymatic activities during sludge and lignocellulosic substrate are recorded for phosphatase and urease enzymes. However, the dehydrogenase activity was slightly stimulated during lignocellulosic composting.

## 4 Discussion

### 4.1 Microbial dynamic during composting

Screening of bacteria, actinobacteria, and fungi is performed during the composting process. The mesophilic microflora was more abundant than the thermophilic (Table 1 and Figure 1). The modifications in the microflora profile during composting depend on the physicochemical and functional variations of the composting substrate. The microbial profile is identical to that shown by Moreno et al. (2021). The microorganism’s dynamic indicates the evolution of composting substrate and indicates their ability to degrade the organic waste. The pathway of microbial metabolism influences the physicochemical parameters during the composting process, which leads to changes in microbial community succession (Chroni et al., 2009; Bhatia et al., 2013; Ge et al., 2020). The high concentration of actinobacteria recorded at the maturation stage is linked to the ability of these strains to grow, and metabolize recalcitrant compounds especially lignocellulosic. Actinobacteria play important ecological roles in the environment, such as degrading complex polymers (Mawang et al., 2021). Actinobacteria are known as potent producers of extracellular enzymes decomposing lignocellulose. Houfani et al. (2022) showed that degrading extracellular enzymes, the actinobacteria display the great ability to decompose cellulose and hemicellulose.

### 4.2 Alkaline phosphatase activity during composting

Phosphatase activity increased markedly at the outset of the composting process of mixture A, reaching maximum activity during the thermophilic stage of the process (Figure 3a), then decreased progressively.

Phosphatase activity in mixture A with a high rate of lignocellulosic bulking agent at the outset of the composting process was markedly higher than that in mixture B (*p* < 0.05) (Figures 2a, 3a). Nevertheless, mixture B is characterized by a homogenous enzymatic activity between different stages during composting. Two-fold higher enzymatic activity was noticed between higher and lowest values for both mixtures A and B (Figure 2a). The enzymatic pattern of expression reflected microbial succession during the composting process and organic matter decomposition. During composting the high phosphatase enzymatic activity was positively correlated with the mesophilic bacteria, and both mesophilic and thermophilic fungi for mixtures A (Table 1). For mixture B, a high correlation was shown for both. Mixture A is characterized by the high rate of recalcitrant compounds due to the proportion of lignocellulosic substrate in the mixture, which could contribute to the dynamic change of microbial activity as well as enzyme activities during the composting process. Several kinds of microorganisms can produce and release large amounts of extracellular phosphatase, due to large, combined biomass, high metabolic activity, and short life cycles (Speir and Ross, 1978; Roy et al., 2004; Jindo et al., 2014). Many fungi are known for their high capacity to produce phosphatases in the form of intracellular and extracellular enzymes (Tarafdar and Marschner, 1995; Cairney, 2011; Sarr et al., 2020). Strains of Bacteria have been studied for their ability to produce phosphatases (Rodríguez and Fraga, 1999; Albrecht et al., 2010; Wan et al., 2020).

The large amounts of carbonaceous substrates incorporated with the lignocellulosic substrate slow the degradation and provide a source of labile carbon to microorganisms. A similar temporal variation of phosphatase activity was observed by Benitez et al. (1999), Tiquia (2002), and Ros et al. (2006). Benitez et al. (1999) explained that this trend seemed to be supported by free PO_4_^−3^ nutrients, which were, in part, transformed into microbial and worm tissues. Phosphatase activity may also depend on the nutrient requirements in the mixtures. The activity of phosphatases depends on the richness of the mixture in Pi, the abundance of which can inhibit the biosynthesis of phosphatases (Pan and Yu, 2011). The positive correlation between Olsen-extractable P and both acid and alkaline phosphatase activities was observed. Nevertheless, phosphatase activity disappeared at critical soluble P of 600–800 mg P/Kg soil (Speir and Ross, 1978).

Indeed, the physical–chemical conditions undergone during composting can influence the microbiological activity and therefore the production of enzymes. Raising the temperature during the composting process to reach 65°C for both mixtures A and B could also influence the expression of enzyme activity. The maximum phosphatase activities have been measured for temperatures of 60–70°C (Antibus et al., 1986; Wannet et al., 2000) or 35°C (Huang et al., 2005). Phosphatase activity could be affected by composting parameters (Ye et al., 2015). When the pH is unfavorable to the enzyme, it modifies its structure and denatures it (Frankenberger and Johanson, 1982). Allison et al. (2007) and Joanisse et al. (2007) showed that phenolic compounds such as tannins and humic acids have inhibitory effects on the phosphatase activity. The adsorption of enzymes on the substrate could also have strong consequences not only on their mobility and activity but also on their stability (George et al., 2007).

### 4.3 Urease activity during composting

Urease activity increased in both mixtures, reaching maximum activity during the thermophilic phase of the composting process. As the composting progressed, the urease activity of each mixture gradually decreased. Then, a second peak of urease enzyme activity increases at the 10th week of the maturation stage (Figure 3b).

Urease enzymatic activity was higher in mixture A than in mixture B (*p* < 0.05) (Figure 2b). However, mixture B is characterized by a homogenous enzymatic activity between different stages during composting (Figure 3b). The positive correlation between urease activity and microorganisms was higher between mesophilic bacteria, and thermophilic and mesophilic fungi, and thermophilic actinobacteria for mixture A (Table 1). For mixture B, the positive correlation was for both mesophilic and thermophilic bacteria and fungi (Figure 3b). That indicates the microbial transformation of organic matter during lignocellulosic sludge composting. The urease activity is due to extracellular enzymes (Xie et al., 2021). Guo et al. (2012) showed that the urease enzyme is principally of microbial origin. The rapid increase in urease activity indicates that nitrogen sources are promptly utilized by the growing microflora (Galli et al., 1997; Liang et al., 2014). The difference in urease activity pattern between the two mixtures could be related to the proportion of substrates of each mixture especially the lignocellulosic one. Ros et al. (2006) suggested that either the greater porosity of the mixture with the bulking agent favored the activity of these enzymes or that the degradation of the bulking agent gave rise to the formation of N substrates susceptible to use by these enzymes.

Urease activity rapidly increased as the composting of the mixture proceeded, reaching its maximum value; then a sharp decrease was recorded after the 10th week. This evolution trend is probably due to the inhibition of the urease system by the ammonia produced (Galli et al., 1997). Nitrogen is necessary for the microbial fermentation of nutrients, especially for protein synthesis and energy, whereas the urease enzyme plays an important role in the nitrogen and carbon cycles (Kumari and Rao, 2017; Zhou et al., 2021). The urease is considered a stress response that was developed by several bacteria to counteract a low environmental pH (Arioli et al., 2007, 2009). Guo et al. (2012) and Kumar et al. (2020) showed that the activity of urease and NH^+^_4_-N removal showed a positive correlation. Under composting conditions, proteins are rapidly hydrolyzed; urease is the key enzyme catalyzing the decomposition of carbamide into NH_3_ and carbonic acid (Meng et al., 2020). Ros et al. (2006) showed the activity of enzymes increased with composting probably due to the disappearance of ammonium, which can act as an inhibitor since it is the product of the hydrolytic reactions catalyzed by these enzymes from the medium and/or to the formation of the specific substrates of these enzymes as organic matter is degraded. The consistent decrease in the urease activity can be attributed to the depletion of readily metabolizable fractions of the organic matter, which presumably support microbial activity (Sudkolai and Nourbakhsh, 2017).

### 4.4 Dehydrogenase activity during composting

The variation trend of dehydrogenase activity in each mixture A and B was basically different over time. During the early stabilization stage of composting, dehydrogenase activity in mixture B, which had a low rate of lignocellulosic substrate, was markedly two-fold higher than that in mixture A (*p* < 0.05). In mixture B, dehydrogenase activity increased rapidly during the 3rd week (thermophilic stage) but then declined substantially thereafter (Figure 3c). Dehydrogenase activity during composting of mixture A showed a continuous upward tendency until the 10th week (maturation stage) and then declined slightly toward the end of the process (Figure 3c). The no reappearance of a second peak during the maturation stage of mixture B explains the high maturity and quality of compost B. Contrary to other enzymes urease and alkaline phosphatase, dehydrogenase activity is lowest in mixture A than B with a homogenous pattern activity between different composting stage (Figures 2c, 3c).

The substrate in mixture A needs more time to be degraded. Dehydrogenase activity during composting was positively correlated with thermophilic actinobacteria, mesophilic bacteria, and both thermophilic and mesophilic fungi for mixture A; and with thermophilic and mesophilic fungi and bacteria for mixture B (Table 1). This sensitivity of dehydrogenase activity could be attributed to the higher concentration of lignocellulosic substrate in mixture A. The initial, high dehydrogenase activity recorded for mixture B might have been the result of the high microbial activity due to the availability of easy metabolizable substrates. The lignocellulosic compounds in mixture A need more time and high microbial activity to be decomposed, which explains the continuous dehydrogenase activity that occurred until the maturation stage. This dehydrogenase activity pattern was like what was observed by Benitez et al. (1999), Barrena et al. (2008), and He et al. (2013).

Actinobacteria and fungi produce an active enzyme to degrade lignocellulosic compounds. The enzymatic capacity of actinobacteria to attack recalcitrant molecules is shown by several authors (Tuomela et al., 2000; El Fels et al., 2014c, 2016; Ijoma and Tekere, 2017). The importance of high fungal enzyme levels for efficient degradation of recalcitrant compounds was better demonstrated (Novotný et al., 2004). Saha et al. (2008) and Ge et al. (2020) showed that dehydrogenase enzymatic activity occurs during composting indicating a high quality of composting microbial activities indicating the degradation of easily available organic substrates. The soluble organic matter in the composted substrates is initially assimilated by the microorganisms; once the soluble organic matter is used up, microorganisms produce hydrolytic enzymes which depolymerize the larger compounds (lignin, cellulose, and hemicellulose) into smaller fragments that are water-soluble (Tate, 1995; Tiquia et al., 2002; Yadav, 2017). Mani et al. (2016) show that the hydrolytic processes result in organic matter decomposition. Dehydrogenase activities reflect the presence of active microorganisms during the composting process (Bohacz, 2018). Benitez et al. (1999) and He et al. (2013) showed that the dehydrogenase activities are sensitive indicators of the state and stabilization of the organic matter.

Dehydrogenase activity corresponds to high microbiological activities, which gradually decrease in the maturation stage. The maturation phase is characterized by a reduction of hydrolytic activities and microbial activity. The stabilization of the dehydrogenase activity would mean that during the early stages, most of the easily available organic matter had been decomposed. Dehydrogenase activity is a general measure of viable microorganisms (Gil-Sotres et al., 2005; Sharma et al., 2017). The intracellular microbial biomass dehydrogenases are a common enzyme and are rapidly degraded following cell death (Kızılkaya, 2008). Hydrogenase activity has been used as a measure of overall microbial activity (García and Hernández, 1997; Poladyan et al., 2019), as it is an intracellular enzyme related to the oxidative phosphorylation process (Trevor et al., 1987; Cook et al., 2017). Garcia et al. (1994) and Khanna et al. (2017) showed that these enzymatic activities are most probably intracellular activities of proliferating microorganisms.

### 4.5 Enzymatic activity trend during lignocellulose-rich mixture to predict compost quality

The enzymes dynamic between both mixtures A and B. Results show that alkaline phosphatase is the most dominant enzymatic activity for both mixtures with a maximum rate of approximately 398.76 and 265.35 μg PNP g^−1^ h^−1^, respectively, for A and B (Figures 2, 5), followed by the urease activity with a maximum of 151.49 and 122.42 μg NH_4_-N g^−1^ 2 h^−1^, respectively, for A and B (Figures 2, 5). Nevertheless, the lowest and most homogenous enzymatic activity was recorded for dehydrogenase activity during composting by only a maximum of approximately 72.29 and 103.28 μg TPF g^−1^ d^−1^, respectively, for A and B (Figures 2, 5).

Except for the dehydrogenase enzyme, the high enzymatic activity for both alkaline phosphatase and urease was shown for mixture A with a higher rate of lignocellulosic carbonic substrates than mixture B. In contrast, higher enzymatic of dehydrogenase was observed for mixture B than for mixture A (Figures 2, 3). In addition to physicochemical parameters, this variation trend between enzyme activities during the composting process is linked especially to the composting substrates, physicochemical parameters, and the kind of functional endogenous microorganisms loaded by the used substrates for composting. Significant changes in the physical and chemical parameters during composting substrates have a significant effect on microflora dynamics and as a consequence on their enzymatic activities (Tables 1, 3 and Figure 4). Composted substrates are key parameters that can affect the enzyme dynamics during the composting process. Significance effects from the explanatory (composting stage, compost mixtures, phosphatase, dehydrogenases, urease activity, bacteria, fungi, and actinobacteria compositions) to predict NH_4_^+^/NO_3_^−^, and C/N ratios, decomposition rate, and HI as response variables from linear mixed models are shown in Table 1. The composting time significantly explained the NH_4_^+^/NO_3_^−^, ratio variation during the compositing and reported a highly significant *p-*value (*F* = −7.2; *p* < 0.001). Similarly, the C/N ratio variability was significantly explained by compositing time (*F* = −10.9; *p* < 0.001), urease activity (*F* = −3.1; *p* < 0.005), and fungi percentage (*F* = 2.3; *p* < 0.004), respectively (Table 1). The composting time (*F* = 9.52; *p* < 0.001) and fungi percentage (*F* = 2.75; *p* < 0.013) explained positively the compost decomposition rate (Table 1).

## 5 Conclusion

The effect of two lignocellulosic proportions was investigated to assess the enzymatic trend during the composting process. Dehydrogenase was the most sensitive enzyme in this study. Dehydrogenase shows a steady increase to reach a peak of 72.29 μg TPF g^−1^ during the maturation stage of lignocellulosic-rich substrate. In contrast, dehydrogenase in mixture B shows a peak during the thermophilic stage of 103.28 μg TPF g^−1^. Dehydrogenase activity was linked to the functional microbial activities; it was positively correlated with thermophilic actinobacteria, mesophilic bacteria, and both thermophilic and mesophilic fungi for mixture A; and with only thermophilic and mesophilic fungi and bacteria for mixture B. Dehydrogenase activities are sensitive indicator of the stabilization of the organic matter and could be used as a good indicator of compost quality. The results of this study confirm that enzymatic activity variation during composting is linked to the physicochemical parameters, functional microbial activities variation, and especially to the composted substrates. These findings are helpful approaches for evaluating the composting progress, indicating the direct usefulness of parameters as indicators of microbial activity and dynamics of the composting maturity.

## Data Availability

The datasets presented in this study can be found in online repositories. The names of the repository/repositories and accession number(s) can be found in the article/supplementary material.
